# Idiopathic megabowel causing acute bowel obstruction: A case report

**DOI:** 10.1016/j.amsu.2022.103665

**Published:** 2022-04-22

**Authors:** Hazem Beji, Fatma Najib, Asma Zaiem, Mohamed Guelbi, Wael Rebai, Montassar Kacem

**Affiliations:** a Department of General Surgery A, Hospital La Rabta, Tunis, Tunisia; b University Tunis El Manar, Faculty of Medicine of Tunis, Tunisia

**Keywords:** Idiopathic megabowel, Idiopathic megacolon, Idiopathic megarectum, Bowel obstruction, Case report

## Abstract

**Background:**

Idiopathic megabowel is a rare condition. Its physiopathology is still unclear. We report a case of a patient presenting with acute bowel obstruction due to idiopathic megacolon and megarectum that was successfully treated with proctocolectomy.

**Case presentation:**

A 65-year-old man, with a history of chronic constipation, presented to the emergency with acute bowel obstruction symptoms.

His CT scan on admission showed a very dilated rectum and sigmoid colon filled with faeces. The patient was managed conservatively.

Due to the deterioration of his condition. We opted for an emergency laparotomy and it revealed the important dilation of the descending colon, the sigmoid colon, and the rectum leading to a Hartmann procedure.

degenerative lesions of the smooth muscle layers were seen on the histopthological report and the diagnosis of rectosigmoid idiopathic megacolon was made.

Postoperatively, we performed a rectoscopy that showed a distended rectum full of faeces.

We performed a proctectomy with colo-anal anastomosis. He had an uneventful recovery.

**Conclusion:**

Idiopathic megacolon is a rare condition. The pathogenesis is still unclear. Surgical treatment is the best option to prevent complications and to improve the quality of life of the patient.

## Highlights

• Idiopathic megabowel is a rare condition.
• It is characterized by a persistent dilatation of the colon and the rectum.
• The physiopathology is still unclear.
• Acute bowel obstruction complicating idiopathic megabowel should be treated timely.
• Surgical treatment is the best option to prevent complications.

## 1 Background

Idiopathic megabowel is a rare condition. Its physiopathology is still unclear. However, It was hypothesized that there are anomalies in the extrinsic and enteric nervous system [1]. It is characterized by a persistent dilatation of the colon and the rectum. It is a diagnosis of exclusion. It manifests as abdominal pain, abnormal bowel movement, and bloating [2]. We report a case of a patient presenting with acute bowel obstruction due to idiopathic megacolon and megarectum that was successfully treated with proctocolectomy.

This case report has been reported in line with the SCARE 2020 Criteria [3].

## 2 Case presentation

A 65-year-old man, with a history of chronic constipation, presented to emergency with abdominal pain, vomiting associated with abdominal distension for one week.

On examination, he had a tender, distended abdomen. Digital rectal examination revealed hard stool impaction. He had normal haemoglobin and leucocytes count. Renal functions were normal.

**Fig. 1:**
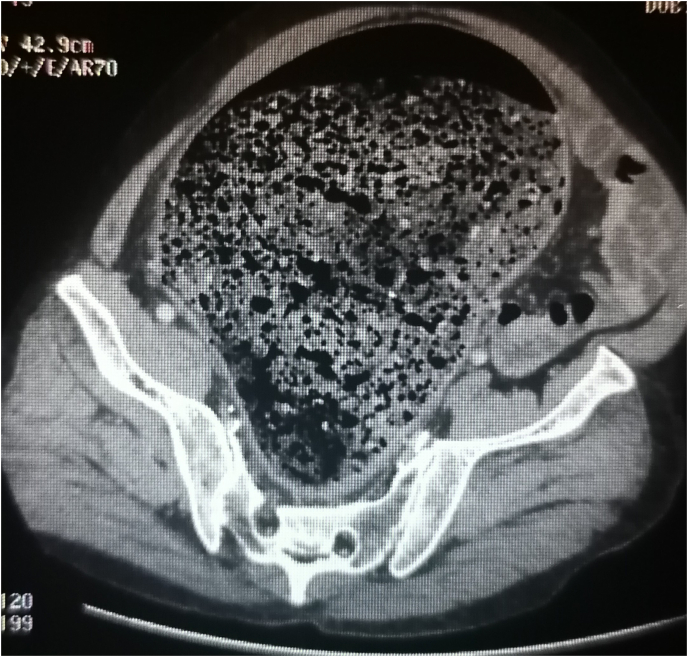
CT scan on admission showing a very dilated sigmoid colon filled with faeces.

His CT scan on admission showed a very dilated rectum and sigmoid colon filled with faeces (Fig. 1). We evoked Hirschprung disease, Ogilvie syndrome, fecloma, and megabowel due to infections or inflammatory bowel disease.

The patient was managed conservatively with oral laxatives and enemas.

His condition was not improving. Three days later, he complained of worsening abdominal pain. He had tachycardia and abdominal tenderness.

**Fig. 2:**
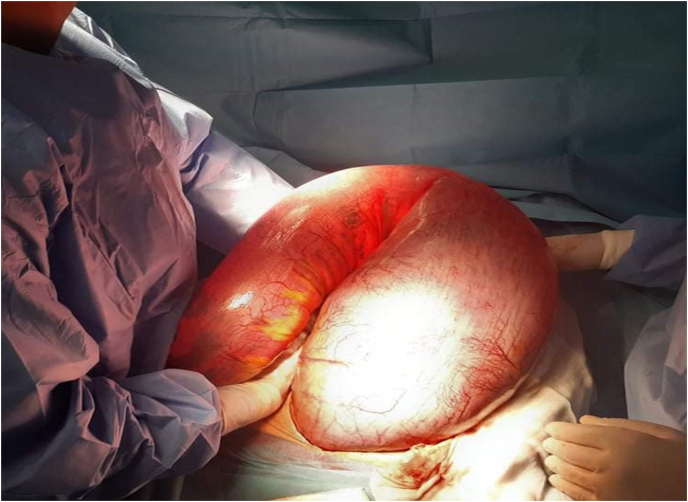
Intra operative view of the mega sigmoid colon.

**Fig. 3:**
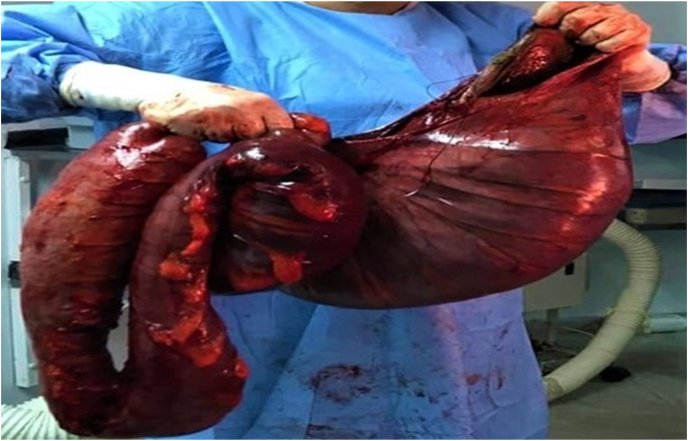
Resection specimen of the left and sigmoid colon.

We opted for an emergency laparotomy. It was performed by a surgeon with sixteen-years experience. It revealed that the descending colon, the sigmoid colon, and the rectum were very dilated (Fig. 2, Fig. 3). The right and the transverse colon were normal. We performed a Hartmann procedure.

He had an uneventful recovery and was discharged after five days.

Histopathology showed degenerative lesions of the smooth muscle layers. There were a normal number of ganglia in the myenteric plexus and no hypertrophic nerve bundle. The diagnosis of idiopathic megabowel was made.

Postoperatively, we performed a rectoscopy that showed a distended rectum full of faeces. Biopsies were done. Histopathology showed no abnormalities.

**Fig. 4:**
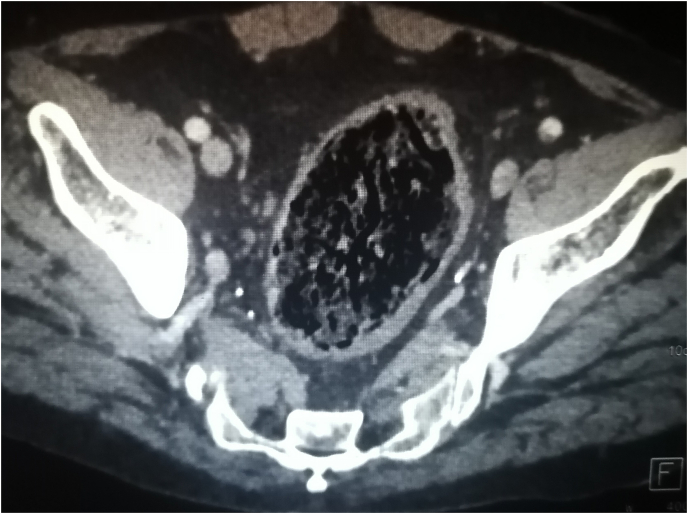
CT scan in the axial plane showing the megarectum after the first surgery.

**Fig. 5:**
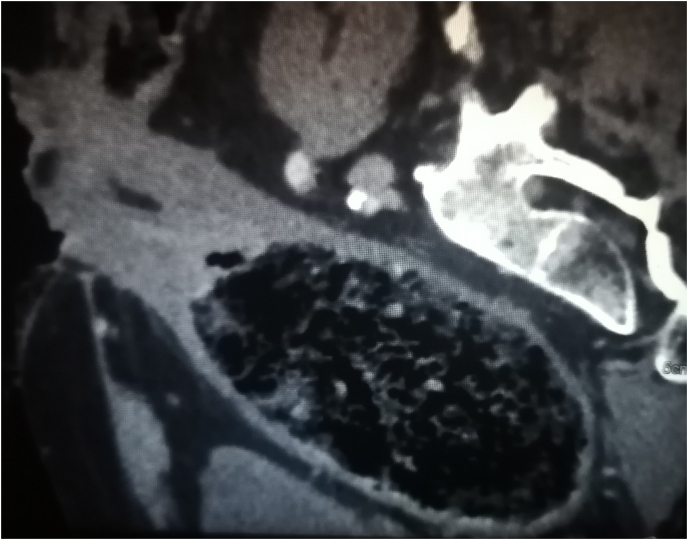
CT scan in the sagittal plane showing the megarectum after the first surgery.

CT scan showed a dilated rectum full of faeces. (Fig. 4, Fig. 5).

He undertook re-laparotomy ten weeks after the first surgery. We performed a proctectomy with colo-anal anastomosis.

He had an uneventful recovery and was discharged home after one week. He had good functional results after eight months of follow-up.

## 3 Discussion

This is one of the rare cases describing idiopathic megacolon and megarectum. We reported successful surgical treatment for this condition.

Our misunderstanding of the pathogenesis of the disease is a weakness of our work. We only treated the consequence of the disease. Further genetic and histopathological studies should be conducted to explain better the pathogenesis of this condition.

Idiopathic megacolon and megarectum is described as important distension of the colon and the rectum in the absence of an identifiable reason. Other causes of megacolon such as Hirschprung disease, medications, infections, inflammatory bowel disease, and autoimmune conditions should be ruled out.

Idiopathic megacolon can be associated with alterations within the gut wall. It was postulated that a decrease in the number of interstitial cells of Cajal (ICCs), regarded as intestinal pacemaker cells, is seen in patients with chronic constipation and megacolon [4,5]. Abnormalities of smooth muscle cells within the bowel can be relevant for the development of megacolon [2].

It was suggested that SEMA3F gene plays a central role in the etiopathogenesis of this pathology [6].

The most frequent symptom is chronic constipation that responds improperly to medical treatment [[7], [8], [9]]. Both genders are affected equally by the disease and symptoms may appear at any age [7].

Early detection of signs of chronic idiopathic megacolon is essential to avoid complications and to perform a more conservative therapeutic strategy.

Acute bowel obstruction due to the stercoral stasis should lead to emergency surgical treatment. Delay of surgery can lead to death. It is most commonly due to a perforation of the dilated bowel and subsequent peritonitis and sepsis or to fluid shift and consequently metabolic and electrolytic abnormalities [10].

Surgical treatment is the only efficient treatment. The different surgical options for idiopathic megabowel are essentially colon and/or rectal resection, pelvic floor procedures, or fecal diversion [7]. Bowel resection is the best option knowing that conservative treatments with oral laxatives and enemas are not efficient [11]. If only a part of the colon is affected, segmental colectomy is sufficient. If the distension affects the rectum, proctocolectomy is then the method of choice [5].

Surgery is more difficult in patients with megarectum [12]. This is explained by the impossibility of adequate bowel preparation. The dilated rectum fills the entire pelvis, making access very challenging. The presence of numerous and dilated rectal vessels makes mobilization hazardous [13].

To date, there are no standardized guidelines for the treatment of idiopathic megabowel. We highlight the importance of timely surgical treatment in presence of acute bowel obstruction. It permits avoiding fatal complications and improving the quality of life of the patient. Further genetic studies with a large sample size should be conducted to better explain the pathogenesis of this condition and to assess standardized guidelines.

## 4 Conclusion

Idiopathic megabowel is a rare condition. The pathogenesis is still unclear. It is a diagnostic of exclusion. Surgical treatment is the best option to prevent complications and to improve the quality of life of the patient.

## Ethical approval

Not required.

## Sources of funding

This research did not receive any specific grant from funding agencies in the public, commercial, or not-for-profit sectors.

## Author contributions

Hazem Beji and Fatma Najib did the conception and design of the work, the data collection, the data analysis and interpretation, and the writing of the manuscript.

Asma Zaiem and Mohamed Guelbi participated in the writing of the manuscript.

Wael Rebai and Montassar Kacem did the critical revision of the article and the final approval of the version to be published.

## Guarantor

Dr Hazem Beji.

Dr Wael Rebai.

## Patient consent

Written informed consent was obtained from the patient for publication of this case report and accompanying images. A copy of the written consent is available for review by the Editor-in-Chief of this journal on request.

## Provenance and peer review

Not commissioned, externally peer reviewed.

## Declaration of competing interest

No conflicts of interest.
